# Vision-Aided RAIM: A New Method for GPS Integrity Monitoring in Approach and Landing Phase

**DOI:** 10.3390/s150922854

**Published:** 2015-09-10

**Authors:** Li Fu, Jun Zhang, Rui Li, Xianbin Cao, Jinling Wang

**Affiliations:** 1 School of Electronic and Information Engineering, Beihang University, Beijing 100191, China; E-Mails: fuli@ee.buaa.edu.cn (L.F.); lee_ruin@ee.buaa.edu.cn (R.L.); xbcao@buaa.edu.cn (X.C.); 2 School of Civil and Environmental Engineering, UNSW Australia, Sydney NSW 2052, Australia; E-Mail: jinling.wang@unsw.edu.au

**Keywords:** GPS, RAIM, aviation navigation, approach and landing phase, computer vision, VA-RAIM

## Abstract

In the 1980s, Global Positioning System (GPS) receiver autonomous integrity monitoring (RAIM) was proposed to provide the integrity of a navigation system by checking the consistency of GPS measurements. However, during the approach and landing phase of a flight path, where there is often low GPS visibility conditions, the performance of the existing RAIM method may not meet the stringent aviation requirements for availability and integrity due to insufficient observations. To solve this problem, a new RAIM method, named vision-aided RAIM (VA-RAIM), is proposed for GPS integrity monitoring in the approach and landing phase. By introducing landmarks as pseudo-satellites, the VA-RAIM enriches the navigation observations to improve the performance of RAIM. In the method, a computer vision system photographs and matches these landmarks to obtain additional measurements for navigation. Nevertheless, the challenging issue is that such additional measurements may suffer from vision errors. To ensure the reliability of the vision measurements, a GPS-based calibration algorithm is presented to reduce the time-invariant part of the vision errors. Then, the calibrated vision measurements are integrated with the GPS observations for integrity monitoring. Simulation results show that the VA-RAIM outperforms the conventional RAIM with a higher level of availability and fault detection rate.

## 1. Introduction

With the wide utilization of satellite navigation technology, the integrity of navigation solutions has become a major issue, especially for safety-of-life applications [1,2,3]. Receiver autonomous integrity monitoring (RAIM) is essential for a GPS receiver, which is developed to assess the integrity of the received GPS signals and provide a timely warning message to the user in the presence of a failure. As an approach and landing phase is a typical part of a flight path with unsafe incidents, designing a suitable RAIM procedure is especially important to guarantee flight safety. Normally, the performance of RAIM relies on a sufficient number of visible satellites and a fine geometrical configuration to check the consistency of the measurements. However, since the approach and landing phase is usually consistent with low GPS visibility conditions due to obstructions, there is a larger mask angle leading to insufficient visible satellites and a poor geometrical configuration. Consequently, the availability and fault detection performance of RAIM will decrease dramatically [4,5,6]. Therefore, it is essential to further investigate the RAIM methods in the approach and landing phase.

When the satellite measurements are insufficient, the conventional RAIM method will not be able to meet the stringent navigation performance requirements in the approach and landing phase. To solve this problem, a logical idea is to bring other aids to provide additional measurements for RAIM. In recent decades, additional navigation devices such as barometric altimeter, inertial navigation system, and other satellite constellations have been considered. For example, the vertical ranging of a barometric altimeter is applied to enrich the navigation measurements for RAIM augmentation [7]. However, the accuracy of the barometric altimeter is susceptible to the quickly atmospheric pressure change, which is very common in the approach and landing phase. With the speeds and attitudes provided by an inertial navigation system (INS), Bhatti *et al.* established the Kalman equations of integrated INS/GPS to improve the performance of RAIM [8], while the error of the INS may drift over the time and contaminate the integrity result. Inspired by the traditional random sample consensus (RANSAC) algorithm in pattern recognition, Schroth *et al.* designed the range consensus (RANCO) algorithm to improve the multi-fault detection result with the assistance of other constellations [9]. Although the RANCO algorithm performs well with sufficient satellites in the simulation, many parameters need to be set empirically. More recently, some augmented RAIM methods without using additional navigation devices are also investigated. Shi *et al.* proposed a new receiver clock bias prediction model based on the discrete grey model to design the clock-aided RAIM method [10], while some factors upon the receiver clock bias series are uncertain. The map-aided integrity method has been investigated for use in intelligent transportation systems [11]. Unfortunately, the performance of availability has not been improved, as the map-aided method gives the user an alert and changes to an alternative navigation system once GPS is unavailable.

Considering these deficiencies above, it is essential to research on utilizing other measurement sources to aid RAIM in approach and landing phase. In this paper, a vision-aided RAIM (VA-RAIM) is proposed to improve the performance of integrity monitoring. As the satellite measurements are insufficient due to the obstructions, our basic idea is to introduce the landmarks, photographed by a vision system, as the pseudo-satellites to enrich the navigation measurements. To aid RAIM, however, the vision measurements applied for RAIM should be accurate and reliable. Although the existing computer vision technologies perform well in vision information extraction, the vision measurements inevitably incur errors due to background interference and platform dithering. Furthermore, the vision errors may be proportional to the length of the light-of-sight as the vision system is inherently an angling system [12]. To ensure the accuracy of the vision system, the received GPS signals are utilized to calibrate the vision measurements, so as to reduce the error of the vision system. Then, the calibrated vision measurements are utilized to expand the GPS measurement equation in order to improve the performance integrity monitoring.

To the best of our knowledge, studies on vision-aided RAIM in the approach and landing phase are quite rare. This paper aims to improve the RAIM performance with the computer vision system in the low GPS visibility condition. In addition, the method in this paper contains some similarity with the vision navigation, while as mentioned by Dusha *et al.* [13], most emphasis in the vision navigation is on positioning where the satellites is unavailable. Actually, there are only a few researches on the vision/GPS integration. For example, Won *et al.* proposed an integrated navigation system that improves the performance of vision-based navigation by integrating the limited GPS measurements in a low GPS visibility condition [14]. Some other applications of the video-based navigation method can be found in [15]. However, they paid comparatively little attention on utilizing the vision system to augment RAIM.

The remainder of this paper is organized as follows. The detail of the proposed VA-RAIM approach is presented in Section 2. Section 3 is the experiments to demonstrate the practical utility of our approach. Finally, the conclusions are shown in Section 4.

## 2. The Proposed VA-RAIM

### 2.1. Overview of VA-RAIM

The baseline RAIM algorithm is mainly composed of two parts, *i.e.*, availability and fault detection. The availability performance is a major concern of RAIM, which ascertains whether the conditions exist to perform fault detection with sufficient power. Fault detection is a safeguard of the correct function of the system and ensures that measurements do not contain significant failures [16].

**Figure 1:**
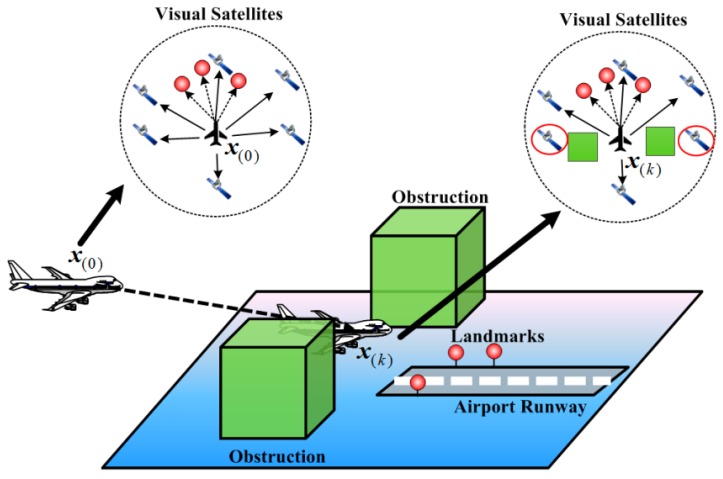
An approach and landing phase scenario.

The availability performance relies on the number of visible satellites and the geometrical configuration. Normally, the RAIM availability requires at least five satellites in view. Besides, the protection level (PL) that is decided by the geometrical configuration should be less than the alert limit (AL) [17]. Unfortunately, in the approach and landing phase, the interferences and obstructions may result in the signal loss and the large mask angle, which will lead to less visible satellites and a poor geometrical configuration. The scenario of an approach and landing phase is shown in Figure 1. In this condition, the availability performance of RAIM will decrease dramatically. A typical example of the approach and landing phase is Chinese LinZhi Airport, which is located at an elevation of 2950 m and flanked by mountains about 5000 m [5]. In some cases, the number of visible satellites is only four, as the satellites with low elevation are blocked by high mountains. When RAIM is available, the RAIM method should detect the system fault in a timely manner to ensure the integrity of the positioning results.

**Figure 2:**
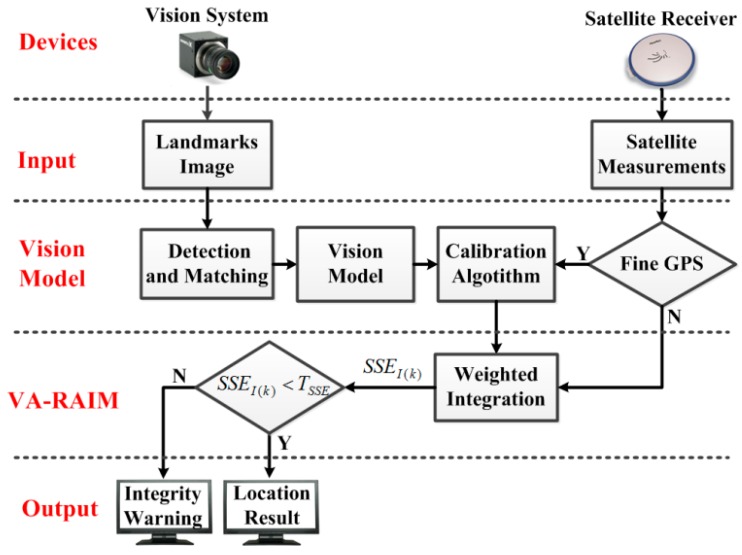
The framework of VA-RAIM.

The purpose of our work is to develop a new RAIM method for the approach and landing phase with the aid of a vision system. To overcome the insufficiency of visible satellites, the VA-RAIM introduces the landmarks photographed by the vision system as pseudo-satellites for additional measurements, as shown in Figure 1. Then, the vision measurements and the GPS observations are integrated to improve navigation integrity. The framework of our approach is shown in Figure 2. To utilize the landmark information with a vision system, the detection and matching algorithm is applied to obtain the landmarks in a given image. However, the image processing will inevitably incur errors due to background interference, platform dithering, *etc.* These errors consist of random time-variant error and time-invariant error, which may be adverse to the integrity performance of the VA-RAIM. To solve this problem, a vision model with the calibration method is proposed. Specifically, the landmarks are introduced as pseudo-satellites and the vision system is modeled as a measurement equation similar to the GPS system. Then, the fine GPS measurements received in high altitude position (such as ***x***_(0)_ in Figure 1) is applied to calibrate the vision pseudoranges to reduce the time-invariant error. Finally, the VA-RAIM is designed by using the weighted integration of the vision and GPS measurements. The test statistics at discrete time *k* of the integrated system *SSE_I_*_(*k*)_ is calculated to compare with a decision threshold *T_SSE_*. If the test statistics is less than the decision threshold, the output will be the location result of the vision/GPS integration. Otherwise, the output will be an integrity warning message. In the proposed method, the vision system is applied as an assistance to enrich the navigation observations and enhance the geometrical configuration, thus improving the performance of GPS integrity monitoring in the approach and landing phase.

### 2.2. Vision Model with Calibration

Under the framework of our approach, the vision system is applied to extract accurate vision pseudoranges from a given image to aid RAIM. Then, a calibration algorithm based on the GPS measurements is designed to reduce the error of the vision observations.

#### 2.2.1. Vision Model

**Figure 3:**
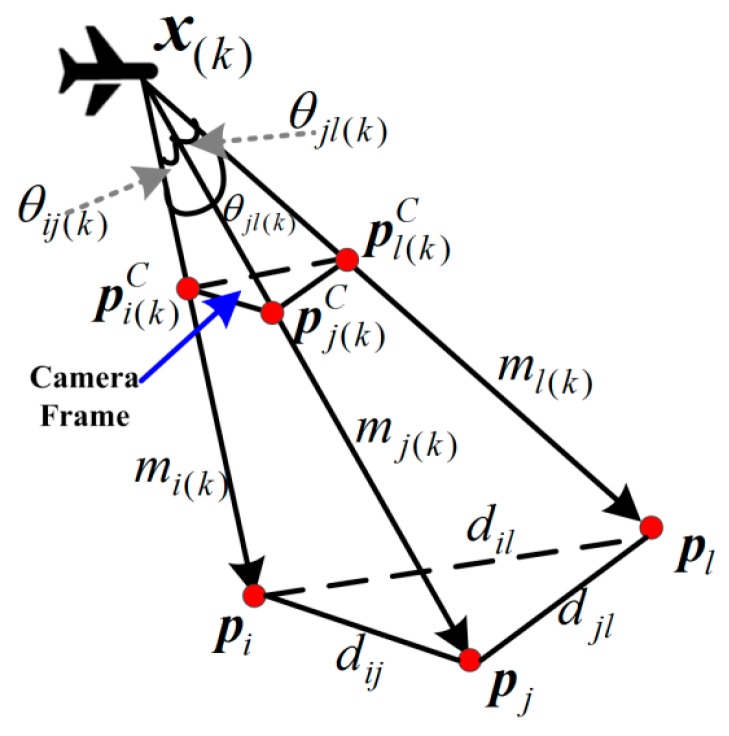
The model of vision pseodoranges.

Inspired by the principle of GPS position model [18], the landmarks are regarded as pseudo-satellites to obtain the similar navigation measurements and a vision pseudorange is defined as the estimation of the distance between the user and a landmark. However, different from GPS pseudoranges, the vision pseudoranges are calculated but directly measured. As shown in Figure 3, at discrete time *k*, denote the vision pseudorange vector ***m***_(*k*)_ = [*m*_1_, *m*_2_, … , $m_{N_{V(k)}}$]^*T*^ ∈ $R^{N_{V(k)}}$, which is composed of *N_V_*_(*k*)_ vision pseudoranges. By applying the cosine rule, the vision pseudorange vector is the solution of the over-determined equation set composed by *N_C_*_(*k*)_=*N_V_*_(*k*)_(*N_V_*_(*k*)_ − 1)/2 equations as:

(1) $$m_{i(k)}^{2}+m_{j(k)}^{2}-2m_{i(k)}m_{j(k)}c_{ij(k)}-d_{ij}^{2}=0,\underset{i(k)\neq j(k)}{\forall }i(k),j(k)\in 1,2,\;...\;,N_{V(k)}$$

where *m_i_*_(*k*)_ ∈ ***R***^+^ is the vision pseudorange of the *i^th^* landmark ***p****_i_* ∈ ***R***^3^ at time *k*. *c_ij_*_(*k*)_ = cosθ*_ij_*_(*k*)_ ∈ [−1, 1], in which θ*_ij_*_(*k*)_ ∈ [0, π] is the angle between the two lines-of-sight of landmarks ***p****_i_* and ***p**_j_* ∈ ***R***^3^. As for the other terms in Equation (1), *d_ij_* = ||***p****_i_* − ***p****_j_*|| ∈ ***R***^+^ is the nominal distance between landmarks ***p****_i_* and ***p****_j_*, which is time-invariant.

(A) Error Analysis

As shown in Equation (1), the vision pseudorange vector is decided by d ij2 and $c_{ij(k)}$. In this section, we take into account *N_V_*_(*k*)_ = 3 to simplify the following analysis, while our approach can also be easily extended to any number of landmarks. The relation between the vision pseudorange error δ***m***_(*k*)_ = [*m*_1(*k*)_, *m*_2(*k*)_, *m*_3(*k*)_]*^T^* and the error of these parameters δ***d*** = [δ$d_{12}^{2}$, δ$d_{23}^{2}$, δ$d_{31}^{2}$]*^T^* and δ***c***_(*k*)_ = [δ*c*_12(*k*)_, δ*c*_23(*k*)_, δ*c*_31(*k*)_]*^T^* can be obtained according to the differential parameters of Equation (1):

(2) $$A_{\left(k\right)}\delta m_{\left(k\right)}=\delta c_{\left(k\right)}+B_{\left(k\right)}\delta d$$

where $A_{\left(k\right)}=\left[\begin{array}{ccc} \frac{1}{m_{2(k)}}-\frac{c_{12(k)}}{m_{1(k)}} & \frac{1}{m_{1(k)}}-\frac{c_{12(k)}}{m_{2(k)}} & 0\\ 0 & \frac{1}{m_{3(k)}}-\frac{c_{23(k)}}{m_{2(k)}} & \frac{1}{m_{2(k)}}-\frac{c_{23(k)}}{m_{3}}\\ \frac{1}{m_{3(k)}}-\frac{c_{31(k)}}{m_{1(k)}} & 0 & \frac{1}{m_{1(k)}}-\frac{c_{31(k)}}{m_{3(k)}} \end{array}\right]$ and $B_{\left(k\right)}=\frac{1}{2m_{1(k)}m_{2(k)}m_{3(k)}}\left[\begin{array}{ccc} m_{3(k)} & 0 & 0\\ 0 & m_{1(k)} & 0\\ 0 & 0 & m_{2(k)} \end{array}\right]$.

Then we can have:

(3) $$\delta m_{\left(k\right)}=A_{\left(k\right)}^{-1}\delta c_{\left(k\right)}+A_{\left(k\right)}^{-1}B_{\left(k\right)}\delta d$$

In Equation (3), since δ***d*** is fixed with a set of given lengths, it generates the time-invariant error of the vision system. δ***c***_(*k*)_ is decided by the detection and matching results at time *k*, which generates the random time-variant error. The properties of δ***d*** and δ***c***_(*k*)_ are analyzed as follows.

If the distance of each pair of landmarks is accurately obtained, we have δ***d*** = **0** and the second part on the right of Equation (3) disappears. However, it is very challenging to measure the distance directly. In practice, we can measure the position of a landmark up to centimeter-level accuracy with the utilization of real-time kinematic (RTK), and then calculate the distance. Specifically, the position difference is calculated as $d_{ij}^{2}$ = ||***p****_i_* − ***p****_j_*||^2^, and the error of the position difference is obtained as δ$d_{ij}^{2}$ = 2(***p****_i_* − ***p****_j_*)*^T^*(δ***p****_i_* − δ***p****_j_*). Suppose the landmark position errors in each degree of freedom obey *N*(0,$\sigma_{g}^{2}$) and independent from each other, δ$d_{ij}^{2}$ obey Gaussian distribution as with zero mean and variance 8$d_{ij}^{2}\sigma_{g}^{2}$, which is proportional to the $d_{ij}^{2}$.

With the transformation projective from object to image space [19], the parameter *c_ij_*_(*k*)_ is calculated by

(4) $$c_{ij(k)}=\langle \frac{p_{i(k)}^{C}}{\left\|p_{i(k)}^{C}\right\|},\frac{p_{j(k)}^{C}}{\left\|p_{j(k)}^{C}\right\|}\rangle$$

where <,·> is the vector inner product, $p_{i(k)}^{C}$ ∈ ***R***^2^ and $p_{j(k)}^{C}$ ∈ ***R***^2^ is the *i^th^* and the *j^th^* landmarks in the camera frame, as shown in Figure 3. The landmarks in the camera frame are obtained through the detection and matching algorithm, and the detection error δ$p_{i(k)}^{C}$ is assumed to follow the independent normal distribution with zero mean and covariance matrix **Σ*_p_*** ∈ ***R***^2×2^.

Linearizing Equation (4) yields:

(5) $$\delta c_{ij(k)}=\langle \delta \frac{p_{i(k)}^{C}}{\left\|p_{i(k)}^{C}\right\|},\frac{p_{j(k)}^{C}}{\left\|p_{j(k)}^{C}\right\|}\rangle +\langle \frac{p_{i(k)}^{C}}{\left\|p_{i(k)}^{C}\right\|},\delta \frac{p_{j(k)}^{C}}{\left\|p_{j(k)}^{C}\right\|}\rangle$$

As proofed in Appendix, δ*c_ij_*_(*k*)_ can be obtained as a linear combination of δ$p_{i(k)}^{C}$ and δ$p_{j(k)}^{C}$, *i.e.*,

(6) $$\delta c_{ij(k)}=\mu_{ij(k)}^{T}\delta p_{i(k)}^{C}+\mu_{ji(k)}^{T}\delta p_{j(k)}^{C}$$

which follows a normal distribution with zero mean and variance $\mu_{ij(k)}^{T}$**Σ_*p*_μ**_*ij*(*k*)_ + $\mu_{ji(k)}^{T}$**Σ_*p*_μ**_*ji*(*k*)_. Thus, we can obtain that δ***c***_(*k*)_ follows a normal distribution with zero mean and covariance matrix **Σ_*c*_**_(*k*)_ ∈ $R^{N_{C(k)}\times N_{C(k)}}$.

(B) Fault Analysis

**Figure 4:**
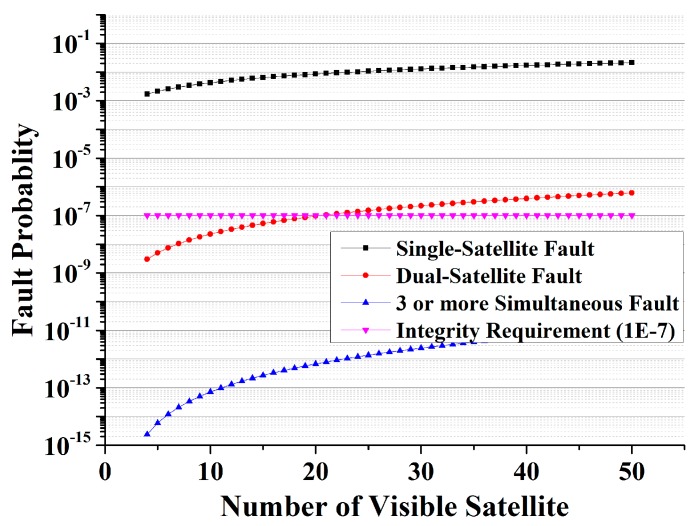
Probabilities of simultaneous satellite faults.

For the RAIM algorithm, a GPS fault is defined as a pseudorange bias deviating from its nominal behavior [2]. The probabilities of simultaneous satellite faults with different number of visible satellite are plotted in Figure 4. Since the scenario of this paper focuses on the approach and landing phase that is a low GPS visibility (often less than 8), the probability of multi-fault simultaneity is less than the integrity requirement 10^−7^ and can be ignored in applications. Thus, the threat of GPS is a fault in at most one visible satellite in this paper. However, if the visible satellites are sufficient that multi-fault cannot be ignored, we can detect the faults by using standalone satellite observations [9].

When used for aiding navigation, vision system may also suffer from faults. With the introduction of vision pseudorange, the fault of vision system ultimately causes a vision pseudorange bias similar to GPS pseudorange bias. As shown in Equation (3), the pseudorange fault is decided by the fault of δ***d*** and δ***c***_(*k*)_. In real applications, there are various types of threats that might cause a fault on δ***d*** or δ***c***_(*k*)_. In this paper, two typical examples are considered as follows, *i.e.*, fault in the landmark location and fault in the feature detection result during image processing.

(1) Fault on δ***d***

If there is a fault Δ***p****_i_* in the location of the *i*^th^ landmark, the error of position difference can be obtained as 2(***p****_i_* − ***p****_j_*)*^T^*(δ***p****_i_* + Δ***p****_i_* − δ***p****_j_*)=δ$d_{ij}^{2}$ + Δ$d_{ji}^{2}$, where Δ$d_{ij}^{2}$ = 2(***p****_i_* − ***p****_j_*)*^T^*Δ***p****_i_* is a bias caused by the location fault. As the location results are prior given, the bias fault is time-invariant during the flight operations. Thus, δ***d*** can be updated to δ***d****′* = δ***d*** + Δ***d***, where Δ***d*** = [Δ$d_{12}^{2}$, Δ$d_{23}^{2}$, Δ$d_{31}^{2}$]*^T^*.

(2) Fault on δ***c***_(*k*)_

If there is a fault Δ$p_{i(k)}^{C}$ in the feature detection result during image processing, combined with Equation (6), δ*c_ij_*_(*k*)_ can be updated to

(7) $$\delta {c_{ij(k)}}^{\prime }=\delta c_{ij(k)}+\mu_{ij(k)}^{T}\Delta p_{i(k)}^{C}+\mu_{ji(k)}^{T}\Delta p_{j(k)}^{C}$$

Denote Δ*c_ij_*_(*k*)_ = $\mu_{ij(k)}^{T}$Δ$p_{i(k)}^{C}$ + $\mu_{ji(k)}^{T}$Δ$p_{j(k)}^{C}$, then δ***c***_(*k*)_ can be updated to δ***c***_(*k*)_*′* = δ***c***_(*k*)_ + Δ***c***_(*k*)_, where Δ***c***_(*k*)_ = [Δ*c*_12(*k*)_, Δ*c*_23(*k*)_, Δ*c*_31(*k*)_]*^T^*.

The probabilities of the vision fault can be statistically obtained with real computer vision data, and the relatively high probabilities often reflect the challenging environment of the data collection, which will be further researched.

(C) Vision Measurement Equation

Similar with the linear GPS measurement equation [20], the linear vision measurement equation with *N_V_*_(*k*)_ landmarks can be modeled as:

(8) $$z_{V(k)}=H_{V(k)}x_{\left(k\right)}+\varepsilon_{V(k)}+b_{V(k)}$$

where ***z***_*V*(*k*)_ ∈ $R^{N_{V(k)}}$ is the vector of vision measurements obtained by the vision pseudoranges at time *k*, ***H***_*V*(*k*)_ ∈ $R^{N_{V(k)}\times 4}$ is the vision observation matrix. ***x***_(*k*)_ ∈ ***R***^4^ is the state vector includes three position elements (*x*_(*k*)_, *y*_(*k*)_ and *z*_(*k*))_ and the receiver clock bias *C_b_*_(*k*)_. **ε**_*V*(*k*)_ ∈ $R^{N_{V(k)}}$ and ***b***_*V*(*k*)_ ∈ $R^{N_{V(k)}}$ are the random error and bias of vision pseudoranges, respectively.

According to Equations (3) and (8), **ε***_V_*_(*k*)_ and ***b**_V_*_(*k*)_ with the consideration of vision fault **Δ*d*** and **Δ*c*_(*k*)_** can be obtained as:

(9) $$\varepsilon_{V(k)}=A_{\left(k\right)}^{-1}\delta c_{\left(k\right)}$$

(10) $$b_{V(k)}=A_{\left(k\right)}^{-1}B_{\left(k\right)}(\delta d\text{+}\Delta d)+A_{\left(k\right)}^{-1}\Delta c_{\left(k\right)}$$

where **ε***_V_*_(*k*)_ follows a normal distribution with zero mean and covariance matrix **Σ***_V_*_(*k*)_ = $A_{\left(k\right)}^{-1}$ **Σ*_c_***_(*k*)_ ${\left(A_{\left(k\right)}^{-1}\right)}^{T}$. According to Equation (10), different with single fault in GPS pseudorange, one fault in Δ***d*** or Δ***c*_(*k*)_** will have an impact in more than one of the vision pseudorange measurements in Equation (8). In the following section, a calibration method is presented to mitigate the impact of the vision faults.

#### 2.2.2. Calibration Method

In this section, the GPS measurements are applied to calibrate the vision system to reduce the error and fault of vision measurements. First, the bias caused by the error and fault of the landmark locations, *i.e.*, δ***d*** and Δ***d***, is removed. Then a modified vision equation with vision single fault is obtained.

With *N_G_*_(*k*)_ GPS satellites in view at discrete time *k*, the linear measurement equation is described as:

(11) $$z_{G(k)}=H_{G(k)}x_{\left(k\right)}+\varepsilon_{G(k)}+b_{G(k)}$$

where ***z**_V_*_(*k*)_ ∈ $R^{N_{G(k)}}$ is the vector of measurements at time *k*, ***H***_*G*(*k*)_ ∈ $R^{N_{G(k)}\times 4}$ is the observation matrix, which reflects the geometrical configuration. **ε**_*G*(*k*)_ ∈ $R^{N_{G(k)}}$ is the measurement noise vector, which are generally assumed to be normally distributed with zero mean and covariance matrix **Σ**_*G*(*k*)_ ∈ $R^{N_{G(k)}\times N_{G(k)}}$. ***b***_*G*(*k*)_ ∈ $R^{N_{G(k)}}$ is the measurement-fault vector to be detected.

At the beginning of the approach and landing phase, such as ***x***_(0)_ in Figure 1, the satellite measurements are sufficient due to little obstruction in the high altitude environment. Under this condition, the fine GPS observations can be obtained and the traditional RAIM is available to compute the trustable positioning results from GPS. Specifically, the GPS positioning solution ${\tilde{x}}_{\left(0\right)}$ ∈ ***R***^4^ obeys normal distribution *N*(***x***_(0)_, **Δ***_G_*_(0)_), where the covariance matrix **Δ***_G_*_(0)_ = ($H_{G(0)}^{T}\;\Sigma_{G(0)}^{-1}\;H_{G(0)}$)^−1^ ∈ ***R***^4 × 4^.

According to Equations (8)–(10), the vision equation at the initial time *k* = 0 and time *k* > 0 is described as:

(12) $$z_{V(0)}=H_{V(0)}x_{\left(0\right)}+A_{\left(0\right)}^{-1}\delta c_{\left(0\right)}+A_{\left(0\right)}^{-1}B_{\left(0\right)}(\delta d+\Delta d)+A_{\left(0\right)}^{-1}\Delta c_{\left(0\right)}$$

and

(13) $$z_{V(k)}=H_{V(k)}x_{\left(k\right)}+A_{\left(k\right)}^{-1}\delta c_{\left(k\right)}+A_{\left(k\right)}^{-1}B_{\left(k\right)}(\delta d+\Delta d)+A_{\left(k\right)}^{-1}\Delta c_{\left(k\right)}$$

Multiply Equation (12) by ($A_{\left(k\right)}^{-1}B_{\left(k\right)}B_{\left(0\right)}^{-1}A_{\left(0\right)}$) and subtract the result from Equation (13) to eliminate the error and fault of landmark locations, and then we have

(14) $$z_{V(k)}=H_{V(k)}x_{\left(k\right)}+A_{\left(k\right)}^{-1}(\delta c_{\left(k\right)}+\Delta c_{\left(k\right)})+A_{\left(k\right)}^{-1}B_{\left(k\right)}B_{\left(0\right)}^{-1}\left(A_{\left(0\right)}z_{V(0)}-\delta c_{\left(0\right)}-\Delta c_{\left(0\right)}\right)-A_{\left(k\right)}^{-1}B_{\left(k\right)}B_{\left(0\right)}^{-1}A_{\left(0\right)}H_{V(0)}x_{\left(0\right)}$$

Substituting $x_{\left(0\right)}={\tilde{x}}_{\left(0\right)}-({\tilde{x}}_{\left(0\right)}-x_{\left(0\right)})$ into Equation (14) yields:

(15) $$z_{V(k)}+A_{\left(k\right)}^{-1}B_{\left(k\right)}B_{\left(0\right)}^{-1}A_{\left(0\right)}(H_{V(0)}{\tilde{x}}_{\left(0\right)}-z_{V(0)})=H_{V(k)}x_{\left(k\right)}+A_{\left(k\right)}^{-1}(\delta c_{\left(k\right)}+\Delta c_{\left(k\right)})+A_{\left(k\right)}^{-1}B_{\left(k\right)}B_{\left(0\right)}^{-1}(A_{\left(0\right)}H_{V(0)}({\tilde{x}}_{\left(0\right)}-x_{\left(0\right)})-\delta c_{\left(0\right)}-\Delta c_{\left(0\right)})$$

Denote

(16) $${\hat{z}}_{V(k)}=z_{V(k)}+A_{\left(k\right)}^{-1}B_{\left(k\right)}B_{\left(0\right)}^{-1}A_{\left(0\right)}(H_{V(0)}{\tilde{x}}_{\left(0\right)}-z_{V(0)})$$

The vision model with calibration is obtained as:

(17) $${\hat{z}}_{V(k)}=H_{V(k)}x_{\left(k\right)}+{\hat{\varepsilon }}_{V(k)}+{\hat{b}}_{V(k)}$$

where ${\hat{\varepsilon }}_{V(k)}=A_{\left(k\right)}^{-1}(\delta c_{\left(k\right)}-B_{\left(k\right)}B_{\left(0\right)}^{-1}\delta c_{\left(0\right)})+A_{\left(k\right)}^{-1}B_{\left(k\right)}B_{\left(0\right)}^{-1}A_{\left(0\right)}H_{V(0)}({\tilde{x}}_{\left(0\right)}-x_{\left(0\right)})$, ${\hat{b}}_{V(k)}=A_{\left(k\right)}^{-1}(\Delta c_{\left(k\right)}-B_{\left(k\right)}B_{\left(0\right)}^{-1}\Delta c_{\left(0\right)})$. It is easy to obtain that ${\hat{\varepsilon }}_{V(k)}$ follows a normal distribution with zero mean and covariance matrix

$${\hat{\Sigma }}_{V(k)}=\Sigma_{V(k)}+A_{\left(k\right)}^{-1}B_{\left(k\right)}B_{\left(0\right)}^{-1}\Sigma_{C(0)}B_{\left(0\right)}^{-1}B_{\left(k\right)}{\left(A_{\left(k\right)}^{-1}\right)}^{T}+A_{\left(k\right)}^{-1}B_{\left(k\right)}B_{\left(0\right)}^{-1}A_{\left(0\right)}H_{V(0)}\Delta_{G(0)}H_{V(0)}^{T}A_{\left(0\right)}^{T}B_{\left(0\right)}^{-1}B_{\left(k\right)}{\left(A_{\left(k\right)}^{-1}\right)}^{T}$$

By comparing the bias vector in Equations (8) and (17), it is illustrated that our method removes the bias caused by the error and fault of the landmark locations. However, the impact of the fault during image processing, *i.e.*, Δ***c*_(*k*)_**, will still exist. According to Equation (17), a fault in Δ***c*_(*k*)_** or Δ***c***_**(**0**)**_ will ripple through all the elements of ${\hat{z}}_{V(k)}$. To mitigate the impacts of Δ***c*_(*k*)_** and Δ***c*_(_**_0_**_)_**, a modified vision equation with single fault is obtained as follows.

In this paper, we assume that there is one fault in Δ***c*_(*k*)_** and the fault index is invariant in a short period of time, *i.e.*, the fault index in Δ***c*_(*k*)_** and Δ***c*_(_**_0_**_)_** are the same. Multiplying Equation (17) by ***A***_(*k*)_ yields a modified vision equation as:

(18) $${\hat{z}}_{V(k)}^{\prime }=H_{V(k)}^{\prime }x_{\left(k\right)}+{\hat{\varepsilon }}_{V(k)}^{\prime }+{\hat{b}}_{V(k)}^{\prime }$$

where ${\hat{z}}_{V(k)}^{\prime }=A_{\left(k\right)}{\hat{z}}_{V(k)}$, $H_{V(k)}^{\prime }=A_{\left(k\right)}H_{V(k)}^{\prime }$, and ${\hat{\varepsilon }}_{V(k)}^{\prime }=\delta c_{\left(k\right)}-B_{\left(k\right)}B_{\left(0\right)}^{-1}\delta c_{\left(0\right)}+B_{\left(k\right)}B_{\left(0\right)}^{-1}A_{\left(0\right)}H_{V(0)}({\tilde{x}}_{\left(0\right)}-x_{\left(0\right)})$,which follows a normal distribution with zero mean and covariance matrix

$${\hat{\Sigma }}_{V(k)}^{\prime }=A_{\left(k\right)}\Sigma_{V(k)}A_{\left(k\right)}^{T}+B_{\left(k\right)}B_{\left(0\right)}^{-1}\Sigma_{C(0)}B_{\left(0\right)}^{-1}B_{\left(k\right)}+B_{\left(k\right)}B_{\left(0\right)}^{-1}A_{\left(0\right)}H_{V(0)}\Delta_{G(0)}H_{V(0)}^{T}A_{\left(0\right)}^{T}B_{\left(0\right)}^{-1}B_{\left(k\right)}$$

In Equation (18), ${\hat{b}}_{V(k)}^{\prime }=\Delta c_{\left(k\right)}-B_{\left(k\right)}B_{\left(0\right)}^{-1}\Delta c_{\left(0\right)}$. According to Equation (2), $B_{\left(k\right)}$ and $B_{\left(0\right)}^{-1}$ are diagonal matrices. Thus, the fault index in **Δ*c*_(*k*)_** and $B_{\left(k\right)}B_{\left(0\right)}^{-1}\Delta c_{\left(0\right)}$ are the same in a short period of time. If there is one fault in **Δ*c*_(*k*)_**, only one fault will exist in the modified observation ${\hat{z}}_{V(k)}^{\prime }$. In the following section, the modified vision equation is integrated with the GPS equation for integrity monitoring.

### 2.3. VA-RAIM

This section presents the VA-RAIM method with the calibrated vision model. We begin with showing the integration of vision/GPS measurement equation. Then the procedure of PL calculation and fault detection of the VA-RAIM is proposed.

By combining Equations (11) and (18), the linearized measurement model at time *k* for the vision/GPS integrated system is obtained as:

(19) $$z_{I(k)}=H_{I(k)}x_{\left(k\right)}+\varepsilon_{I(k)}+b_{I(k)}$$

where $z_{I(k)}=\left[\begin{array}{c} z_{G(k)}\\ {\hat{z}}_{V(k)}^{\prime } \end{array}\right]$, $H_{I(k)}=\left[\begin{array}{c} H_{G(k)}\\ H_{V(k)}^{\prime } \end{array}\right]$, $\varepsilon_{I(k)}=\left[\begin{array}{c} \varepsilon_{G(k)}\\ {\hat{\varepsilon }}_{V(k)}^{\prime } \end{array}\right]$, $b_{I(k)}=\left[\begin{array}{c} b_{G(k)}\\ {\hat{b}}_{V(k)}^{\prime } \end{array}\right]$. The random error **ε**_*I*(*k*)_ follows a normal distribution with zero mean and covariance matrix $\Sigma_{I(k)}=\left[\begin{array}{cc} \Sigma_{G(k)} & 0\\ 0 & {\hat{\Sigma }}_{V(k)}^{\prime } \end{array}\right]$.

With the aid of vision measurements, the over-determined integrated vision/GPS system can improve the integrity performance in the approach and landing phase.

#### 2.3.1. Protection Level and Availability

Protection level computation is in essence a performance safeguard to evaluate the power of fault detection. Denote *N_I_*_(*k*)_ = *N_G_*_(*k*)_ + *N_V_*_(*k*)_ is the sum number of satellites and landmarks, *H_slope_*(*i*) and *V_slope_*(*i*) (*i =* 1,2, …, *N_I_*_(*k*)_) of the integrated system is obtained as [21]:

(20) $$Hslope(i)=\frac{\sqrt{A_{1i}^{2}+A_{2i}^{2}}}{\sqrt{S_{ii}}}$$

(21) $$Vslope(i)=\frac{\left|A_{3i}\right|}{\sqrt{S_{ii}}}$$

where *A*_1*i*_, *A*_2*i*_, and *A*_3*i*_ are the elements of matrix ***A***_*I*(*k*)_ = ($H_{I(k)}^{T}\Sigma_{I(k)}^{-1}H_{I(k)}{)}^{-1}H_{I(k)}^{T}\Sigma_{I(k)}^{-1}$, *S_ii_* is the *i^th^* diagonal elements of matrix ***S***^*I*(*k*)^ = $I_{N_{I(k)}}$ − $H_{I(k)}$ ***A***_*I*(*k*)_, $I_{N_{I(k)}}$ is a *N_I_*_(*k*)_ by *N_I_*_(*k*)_ identity matrix.

Then the calculation of horizon/vertical PL (HPL/VPL) is given as

(22) $$HPL=\max(\text{H}slope(i))T(N_{I(k)},P_{FA})+k(P_{MD})\sqrt{J_{11}+J_{22}}$$

(23) $$VPL=\max(Vslope(i))T(N_{I(k)},P_{FA})+k(P_{MD})\sqrt{J_{33}}$$

where *T*(*N_I_*_(*k*)_, *P_FA_*) is a threshold value, which is a function of *N_I_*_(*k*)_ and the probability of false alarm (FA) *P_FA_*, and follows a chi-square distribution with *N_I_*_(*k*)_ − 4 degrees of freedom. *k*(*P_MD_*) is the number of standard deviations corresponding to a specified probability of missed detection (MD) *P_MD_*. *J*_11_, *J*_22_, and *J*_33_ are the diagonal elements of matrix **Δ***_I_*_(*k*)_ = $(H_{I(k)}^{T}\;\Sigma_{I(k)}^{-1}\;H_{I(k)}{)}^{-1}$.

If the calculated HPL/VPL is larger than the horizon/vertical alert limit (HAL/VAL), *i.e.*, *HPL* > *HAL* or *VPL* > *VAL*, the system is not available and an alert is provided to the user. Otherwise, the fault detection procedure will be processed as follows.

#### 2.3.2. Fault Detection

The fault detection method can be classified as snapshot method [22,23] or filtering method [24]. The former is generally evaluated with the current observations only, while the latter uses both the current and historical measurements. Compared with the filtering method, the snapshot scheme is more widely used due to its faster response to sudden failures [25,26]. Given the measurement Equation (19), the weighted least-squares solution for the estimation of ***x***_(*k*)_ is given by:

(24) $${\tilde{x}}_{\left(k\right)}=A_{I(k)}z_{I(k)}$$

Then, the residual vector is defined as:

(25) $$r_{I(k)}=S_{I(k)}(\varepsilon_{I(k)}+b_{I(k)})$$

The test statistic of the snapshot RAIM at discrete time *k* is given by:

(26) $$SSE_{I(k)}=r_{I(k)}^{T}\Sigma_{I(k)}^{-1}r_{I(k)}$$

As discussed, e.g., in [27], it is well known that the test statistic *SSE_I_*_(*k*)_ follows a noncentral chi-squared distribution with *N_I_*_(*k*)_ − 4 degrees of freedom and noncentrality parameter $\lambda_{I(k)}^{2}\;=\;b_{I(k)}^{T}\;\Sigma_{I(k)}^{-1}S_{I(k)}b_{I(k)}$. The integrity warning is outputted when *SSE_I_*_(*k*)_ is larger than a detection threshold *T*(*N_I_*_(*k*)_, *P_FA_*). Or, it will output the position ${\tilde{x}}_{\left(k\right)}$ if *SSE_I_*_(*k*)_ is less than *T*(*N_I_*_(*k*)_, *P_FA_*).

## 3. Numerical Experiments and Discussions

In this paper, three separate numerical experiments were designed to evaluate the performances of the proposed approach. The first experiment is to assess the performance of the vision pseudorange with calibration. The second experiment is to test the availability result with the aid of vision system. The final experiment is to evaluate the fault detection performance of the VA-RAIM compared with the conventional GPS RAIM algorithm.

### 3.1. Simulation Data

Since the real flying data in the approach and landing phase are very difficult to obtain, simulation data were applied to evaluate the performance of our method. The simulation data are described as follows.

#### 3.1.1. GPS System

In the simulation, the 24 satellite GPS constellation was used to simulate the satellite observations. The peseudorange noises follow the same uncorrelated Gaussian distribution and the diagonal covariance matrices **Σ***_G_*_(*k*)_ satisfies

(27) $$\Sigma_{G(k)(i,i)}=\sigma_{URA(k),i}^{2}+\sigma_{tropo(k),i}^{2}+\sigma_{user(k),i}^{2}$$

where **Σ***_G_*_(*k*)(*i*,*i*)_ is the *i^th^* diagonal elements of **Σ***_G_*_(*k*)_, σ*_URA_*_(*k*),*i*_ is the user range accuracy (URA) of the *i^th^* satellite at time *k*, which is set to be 0.75 m [28]. The error model for tropospheric delay error σ*_tropo_*_(*k*),*i*_ is defined by:

$$\sigma_{tropo(k),i}=0.12\times 1.001/\sqrt{0.002001+\sin{\left(\theta_{i}(k)\right)}^{2}}$$

where θ*_i_*(*k*) is the elevation angle of the *i^th^* satellite. As for the other terms in Equation (27), the user error σ*_user_*_(*k*),*i*_ is modeled according to [29] as:

$$\sigma_{user(k),i}=\sqrt{\sigma_{MP}^{2}+\sigma_{Noise}^{2}}\sqrt{f_{L1}^{4}+f_{L5}^{4}}/（f_{L1}^{2}-f_{L5}^{2}）$$

where $\sigma_{MP}\text{=}0.13\text{+}0.53\exp(-18\theta_{i}(k)/\pi )$, $\sigma_{Noise}\text{=}0.15\text{+}0.43\exp(-180\theta_{i}(k)/6.9/\pi )$, *f_L_*_1_ = 1575.42 MHz, and *f_L_*_5_ = 1176.45 MHz. The bias of the faulty pseudorange was manually set to assert the performance of integrity monitoring.

#### 3.1.2. Vision System

**Figure 5:**
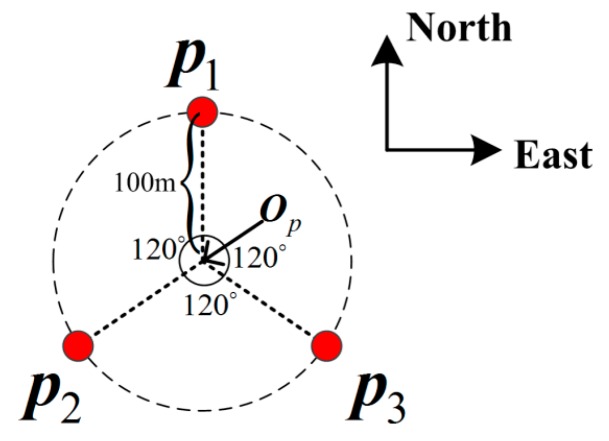
The positions of the landmarks.

Three landmarks were generated with a fixed height at Chinese LinZhi airport. They were evenly distributed on a circle with a center point ***O****_p_ =* [90.3359°, 29.3065°, 2950 m]*^T^* in the Longitude Latitude Height (LLH) frame, and a radius of 100 m. As shown in Figure 5, the azimuths of the landmarks ***p***_1_, ***p***_2_, ***p***_3_ relative to the center point ***O****_p_* are 0°, 120°, and 240°, respectively. To simulate the obstructions, two mountain chains with unified heights of 1000 m are symmetrically located in the 3000 m east and the 3000 m west of the center point.

As shown in Equation (3), the error of vision peseudorange is decided by the landmark position error (LPE) and the feature detection error (DE). In our experiment, different value of LPE and DE were set to evaluate the performance of vision system. LPE was assumed to be centimeter-level that varies from 1 cm to 10 cm (zero LPE is an ideal scenario). DE was set as a white Gaussian distribution [30] with a standard deviation of 1 pixel to 4 pixels on an image with resolution of 120 dpi, 300 dpi, 480 dpi and 720 dpi, where dpi is a unit of image resolution, which means the number of pixels per inch.

#### 3.1.3. Approach and Landing Operations

The length of approach and landing phase was set as 6000 m, which starts from the point [90.2539°, 29.3065°, 3650 m]*^T^* in the LLH frame and ends at point [90.3144°, 29.3065°, 3150 m]*^T^* in the LLH frame. The total length of the simulation date is 6 × 10^6^ s, which is composed by 10^5^ sorties and 60 s for each sortie.

### 3.2. Vision Pseudorange with Calibration

**Figure 6:**
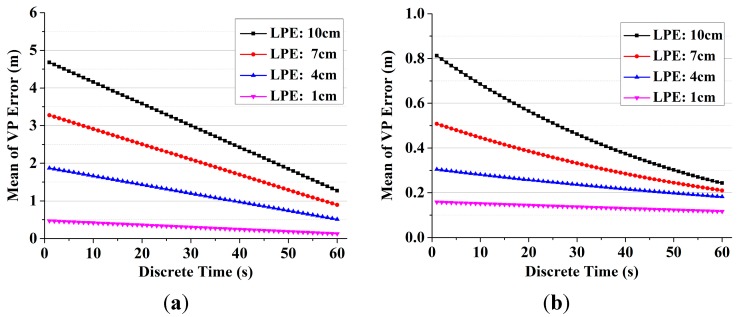
The mean of VP error with different LPE. (**a**) The mean of VP error without calibration; (**b**) The mean of calibrated VP error.

The mean of VP error with different LPE is shown in Figure 6. As shown in Figure 6a, there is a nonzero value range from 4.7 m to 0.2 m in the mean of VP error without calibration. Although the landmark positions are very accurate and LPE is centimeter-level, LPE still causes VP error that cannot be ignored and may raise a false alarm for integrity monitoring. Besides, as the vision system is an angling system, the error caused by LPE approximate linear increases as the length of the line-of-sight increases. As shown in Figure 6b, with the calibration algorithm, the mean of the calibrated VP error is less than 1 m during the simulation. The results illustrate that our calibration algorithm reduces the mean of VP errors significantly. Theoretically, the mean of the calibrated VP error is 0 as shown in Equation (14), while the error cannot be completely reduced due to the linearization error.

**Figure 7:**
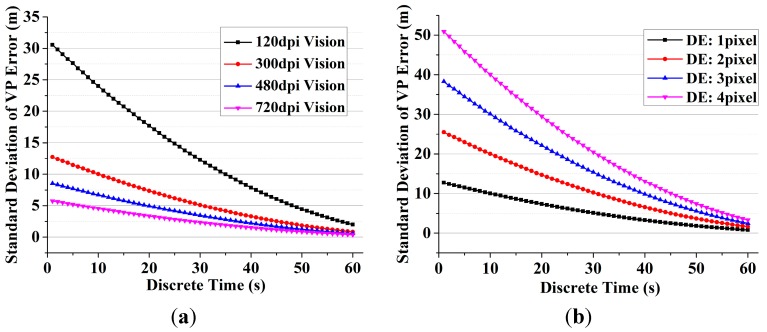
The standard deviation of VP error with different image resolution and DE. (**a**) The standard deviation of VP with different image resolutions with 1 pixel DE; (**b**) The standard deviation of VP with different DE on a 300 dpi image.

The standard deviation of VP error with different image resolution and DE is shown in Figure 7. The results show that higher resolution, shorter line-of-sight and less DE can generate more accurate vision pseudoranges, while the error of vision system is still much larger than GPS system. Such a situation may be improved as feature matching accuracy has been increasing over the years [31].

Although the time-variant error in the vision measurements cannot be protected against directly, we can mitigate its impact by increasing the image resolution and improving the accuracy or robustness of the feature detection algorithm, which is also an important topic in the computer vision community. Furthermore, a consistency check method for the standalone vision system can be applied to reduce the impact of vision fault [12]. The image processing for aviation applications is worth being investigated in our future work.

### 3.3. Performance Index

The performances index is defined with respect to the level of service that the system is designed to provide. The performance requirements for typical approach operations are shown in Table 1, including HAL/VAL, horizon/vertical accuracy (HA/VA (95%)) and time to alert (TTA) [32,33].

**Table 1:** The performance requirements for aviation.

Performance Requirement	APV-I	LPV-200	APV-II
HAL	40 m	40 m	40 m
VAL	50 m	35 m	20 m
HA (95%)	16 m	16 m	16 m
VA (95%)	20 m	4 m	8 m
TTA	10 s	6.2 s	6 s

#### 3.3.1. Availability

**Figure 8:**
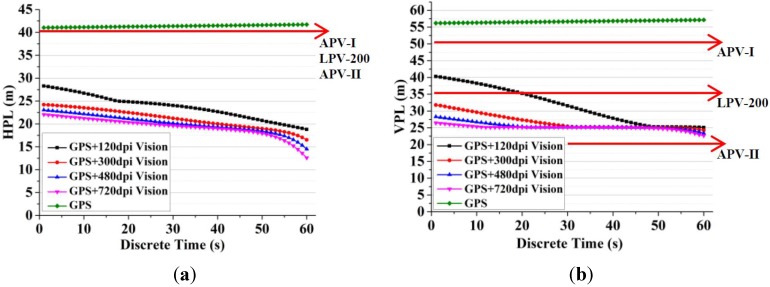
(**a**) The result of HPL with 1 pixel DE; (**b**) The result of HPL with 1 pixel DE.

To evaluate the availability improvement provided by the vision system, the HPL/VPL is computed during the approach and landing phase. The availability of these two methods is calculated and compared with the service levels in Table 1. If the HPL exceeds the HAL or the VPL exceeds the VAL, the integrity is said to be unavailable for operation. The HPL/VPL curves of one sortie are shown in Figure 8. The results show that with the aid of vision system, the HPL decreases from 41 m to 12–26 m, and the VPL decreases from 56 m to 22–40 m, and higher image resolutions can generate less protection level of the integrated system. The reason is that the proposed VA-RAIM provides navigation measurements which are integrated with the GPS measurements to improve availability. Furthermore, since the line-of-sight of vision system is below the aircraft, the vision measurements can improve the geometrical configuration and decrease the protection level. During approach and landing operations, with an accurate feature detection result on a high resolution image, the VA-RAIM can improve the availability performance for APV-I and LPV-200 applications. However, as the performance requirement is very stringent for APV-II, the method needs to be further investigated in the future.

#### 3.3.2. Position Accuracy

Besides the availability requirements, HA/VA (95%) requirements for aviation position during typical operations are shown in Table 1. The horizon error (95%) and vertical error (95%) of our method are 7.1 m and 4.3 m, respectively, and the error (95%) of standalone GPS navigation in horizon and vertical are 8.0 m and 5.2 m, respectively. The position results show that our method can improve the accuracy and meet the HA/VA (95%) requirements of APV-I and APV-II. Recently, vision-aided positioning is being discussed for aviation applications [15], while in this paper we pay more attention to researching integrity during the approach and landing phase.

#### 3.3.3. Time Cost

TTA is another important performance index, as shown in Table 1. The VA-RAIM takes an average of 10 ms in the simulation, since it only contains some fundamental matrix operations except image processing. In real applications, the image processing will be a major part of the time cost. Thus, some efficient feature detection methods would be applied for VA-RAIM to meet the TTA requirement, e.g., scale invariant feature transform (SIFT), which has been proved to be very effective and low time cost for object tracking. For example, the time cost of SIFT for an image with size (pixel × pixel) of 256 × 256 and 441 × 552 are 1.7 ms and 4.4 ms, respectively [34].

### 3.4. Fault Detection

#### 3.4.1. GPS Fault

**Figure 9:**
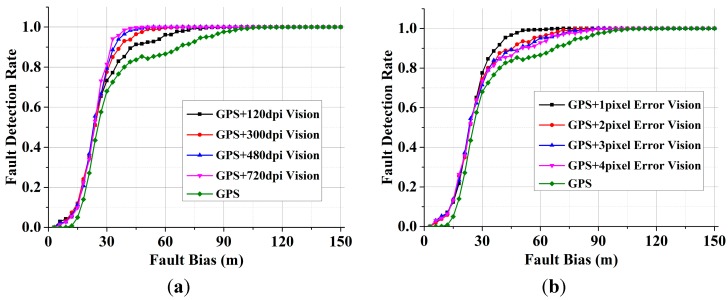
Fault detection rate of GPS RAIM and VA-RAIM. (**a**) GPS RAIM and VA-RAIM with different resolutions with 1 pixel DE; (**b**) GPS RAIM and VA-RAIM with different DE on a 300 dpi image.

To evaluate the performance of our approach, the GPS RAIM and the VA-RAIM were compared in terms of fault detection. We randomly selected one visual satellite and added the fault bias on the pseudorange. The fault detection results with different methods are shown in Figure 9, which shows that the proposed VA-RAIM performs better than the GPS RAIM method with a higher fault detection rate under the conditions of the same fault. For example, when the fault bias is 50 m, the fault detection rate of the GPS RAIM is 84.3%, and the fault detection rate of VA-RAIM with 300 dpi and 1 pixel DE is 97.5%.The VA-RAIM has an increase 13.2% when compared with the fault detection result of the GPS RAIM. Taking into account that the detection power is 99%, the minimal detectable bias (MDB) [16] of the VA-RAIM with 300 dpi and 2 pixels DE is 75 m, which is 21.9% less than the GPS RAIM’s 96 m. Conclusively, the proposed VA-RAIM outperforms the GPS RAIM with a higher level of fault detection rate and lower MDB in the approach and landing phase.

#### 3.4.2. Vision Fault

**Figure 10:**
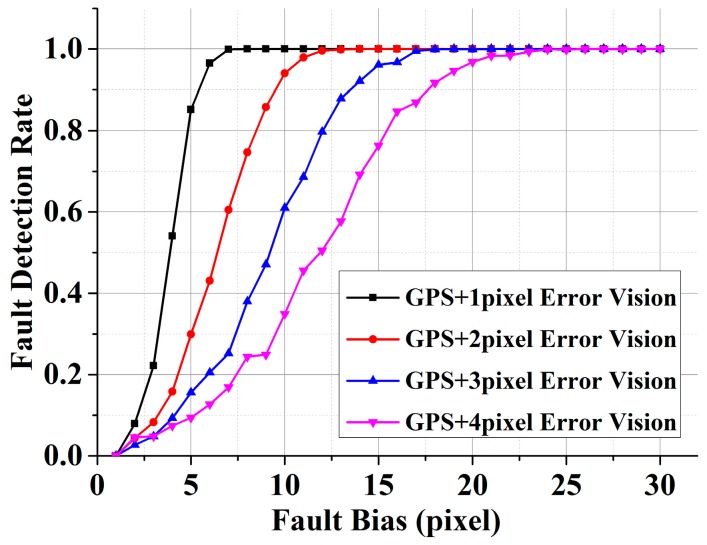
Fault detection rate of vision system with different DE on a 300 dpi image.

In addition, the VA-RAIM may also consider any potential faults within the visual measurements. In this paper, the vision fault is defined to be a fault bias on one of the feature detection results. The fault detection results with different DE on a 300 dpi image are shown in Figure 10. The experimental results show that the vision/GPS integration system can detect the fault of the vision system effectively. With more accurate DE, the method can obtain higher fault detection rate of the vision system, and future research will cover the protection levels associated with visual measurement faults.

## 4. Conclusions

In this paper, we have proposed a VA-RAIM for GPS integrity monitoring in approach and landing phase. To solve the problem that the GPS signals are insufficient for RAIM in approach and landing phase, in the proposed method, the vision system has been used to assist in enriching the navigation observations and geometrical configuration. First, a vision model with the calibration method has been presented to reduce the invariant error of the vision system. Then, the calibrated vision measurements were integrated with the GPS observations to improve the performance of integrity monitoring in the approach and landing phase. Experimental results have demonstrated the effectiveness of the VA-RAIM over the conventional RAIM in availability and fault detection rate.

In addition, the vision system might be limited during night, fog, rain and snow, *etc.*, which may have a large negative impact on the performance of VA-RAIM. To solve this problem, a more powerful feature detection method is worth being investigated. Since the primary concern of this paper is the measurements provided by the vision system rather than the imaging process itself, the location of landmarks in the camera frame is simulated with a Gaussian noise. The future work is to evaluate the practical utility of our VA-RAIM with real data in various scenarios.

## Appendix

Given a vector ***p***, we have

(A1) $$\delta \frac{p}{\left\|p\right\|}=\frac{\delta p\left\|p\right\|-p\delta \left\|p\right\|}{{\left\|p\right\|}^{2}}$$

Substituting δ*||**p***|| = ***p****^T^*δ***p***/||***p***|| into Equation (A1) yields,

(A2) $$\delta \frac{p}{\left\|p\right\|}=\frac{p^{T}p\delta p-pp^{T}\delta p}{{\left\|p\right\|}^{3}}$$

Denote ***p**=r*[cosθ, sinθ]*^T^* ∈ ***R***^2^, we can obtain

(A3) $$\delta \frac{p}{\left\|p\right\|}=\frac{1}{r}\delta p-\frac{1}{r}\left[\begin{array}{cc} \cos^{2}\theta & \cos\theta \sin\theta \\ \cos\theta \sin\theta & \sin^{2}\theta \end{array}\right]\delta p=\frac{1}{r}\left[\begin{array}{cc} \sin^{2}\theta & -\cos\theta \sin\theta \\ -\cos\theta \sin\theta & \cos^{2}\theta \end{array}\right]\delta p$$

According to Equation (A3), denote $p_{i(k)}^{C}$ = *r_i_*_(*k*)_[cosθ*_i_*_(*k*)_, sinθ*_i_*_(*k*)_]*^T^* and $p_{J(k)}^{C}$ = *r_j_*_(*k*)_[cosθ*_j_*_(*k*)_, sinθ*_j_*_(*k*)_]*^T^*, Equation (5) in Section 2.2.1 can be obtained as

(A4) $$\begin{array}{l} \delta c_{ij(k)}={\left(\frac{p_{j(k)}^{C}}{\left\|p_{j(k)}^{C}\right\|}\right)}^{T}\delta \frac{p_{i(k)}^{C}}{\left\|p_{i(k)}^{C}\right\|}+{\left(\frac{p_{i(k)}^{C}}{\left\|p_{i(k)}^{C}\right\|}\right)}^{T}\delta \frac{p_{j(k)}^{C}}{\left\|p_{j(k)}^{C}\right\|}=\\ {}[\cos\theta_{j(k)},\sin\theta_{j(k)}]\frac{1}{r_{i(k)}}\left[\begin{array}{cc} \sin^{2}\theta_{i(k)} & -\cos\theta_{i(k)}\sin\theta_{i(k)}\\ -\cos\theta_{i(k)}\sin\theta_{i(k)} & \cos^{2}\theta_{i(k)} \end{array}\right]\delta p_{i(k)}^{C}+\\ {}[\cos\theta_{i(k)},\sin\theta_{i(k)}]\frac{1}{r_{j(k)}}\left[\begin{array}{cc} \sin^{2}\theta_{j(k)} & -\cos\theta_{j(k)}\sin\theta_{j(k)}\\ -\cos\theta_{j(k)}\sin\theta_{j(k)} & \cos^{2}\theta_{j(k)} \end{array}\right]\delta p_{j(k)}^{C} \end{array}$$

Define $\mu_{ij(k)}=\frac{1}{r_{i(k)}}\left[\begin{array}{cc} \sin^{2}\theta_{i(k)} & -\cos\theta_{i(k)}\sin\theta_{i(k)}\\ -\cos\theta_{i(k)}\sin\theta_{i(k)} & \cos^{2}\theta_{i(k)} \end{array}\right]\left[\begin{array}{c} \cos\theta_{j(k)}\\ \sin\theta_{j(k)} \end{array}\right]$, we have

(A5) $$\delta c_{ij(k)}=\mu_{ij(k)}^{T}\delta p_{i(k)}^{C}+\mu_{ji(k)}^{T}\delta p_{j(k)}^{C}$$
